# Meteorologic Influences on *Plasmodium falciparum* Malaria in the Highland Tea Estates of Kericho, Western Kenya

**DOI:** 10.3201/eid0812.020077

**Published:** 2002-12

**Authors:** G. Dennis Shanks, Simon I. Hay, David I. Stern, Kimutai Biomndo, Robert W. Snow

**Affiliations:** * U.S. Army Medical Research Unit–Kenya, Nairobi, Kenya; † University of Oxford, Oxford, U.K.; ‡ Kenya Medical Research Institute/Wellcome Trust Collaborative Programme, Nairobi, Kenya; § Rensselaer Polytechnic Institute, Troy, New York, USA; ¶ Brooke Bond Central Hospital, Kericho, Kenya

**Keywords:** malaria, epidemiology, highland, meteorology, climate change, global warming

## Abstract

Recent epidemics of *Plasmodium falciparum* malaria have been observed in high-altitude areas of East Africa. Increased malaria incidence in these areas of unstable malaria transmission has been attributed to a variety of changes including global warming. To determine whether the reemergence of malaria in western Kenya could be attributed to changes in meteorologic conditions, we tested for trends in a continuous 30-year monthly malaria incidence dataset (1966–1995) obtained from complete hospital registers at a Kenyan tea plantation. Contemporary monthly meteorologic data (1966–1995) that originated from the tea estate meteorologic station and from global climatology records were also tested for trends. We found that total hospital admissions (malaria and nonmalaria) remained unchanged while malaria admissions increased significantly during the period. We also found that all meteorologic variables showed no trends for significance, even when combined into a monthly suitability index for malaria transmission. We conclude that climate changes have not caused the highland malaria resurgence in western Kenya.

Highland malaria has returned to the tea estates of western Kenya after an absence of nearly 30 years (1–3). Altitude and weather influence malaria epidemiology in highland areas because of the slowing of parasite development within the anopheline vectors at lower temperatures (4). Increased malaria incidence in unstable transmission areas has been variously attributed to changes in land-use patterns (5); population migration (6,7); changes in mosquito vector populations (8); breakdown in provision of health services (9), especially insecticide spraying (10,11); drug resistance (12–16); and meteorologic changes (17,18), particularly global warming (19–25).

We investigated whether climate changes could be implicated in the reemergence of malaria in a unique 30-year malaria and meteorologic time series, collected from the health-care system on a tea plantation in the western highlands of Kenya. Our detailed substudy included site-specific meteorologic and malariometric data from a larger analysis of trends in meteorologic conditions across East Africa from 1911 to 1995 (26–28). Our previous studies have also examined various aspects of the epidemiology of malaria in the Kenyan highlands (29,30).

## Methods

### Study Site and Clinical Data

Long-term malaria illness and total hospital admissions data (January 1966–December 1995) exist from a large tea plantation in Kericho, Kenya, which is operated by Brooke Bond Kenya Ltd. (3,31,32). The plantation, located in the western Rift Valley highlands, covers an area of approximately 141 km^2^ and ranges from 1,780 to 2,225 m above mean sea level. Epidemic malaria was first recorded on the Kericho tea estates during World War II and was eventually controlled by a combination of mass administration of proguanil and residual insecticide spraying during the late 1940s (2). Currently, the Brooke Bond Kenya Ltd. plantation consists of approximately 20 separate tea estates with a total of 50,000 employees and dependents, who receive their medical care from the company-operated health systems. The company hospital maintains a 24-hour, 7-day clinical admission service for patients who need intensive clinical management. Stained blood smears from patients with suspected malaria are examined; this procedure, in combination with further supportive clinical and laboratory procedures, is used to confirm a primary malaria diagnosis. Case numbers in Kericho can be treated as incidence figures since the population eligible for health care remained at approximately 50,000 during the recording period (32). No centralized preventive chemoprophylaxis, vector control, or bed-net distribution has been implemented since the late 1950s. A substantial minority of the tea estate workers originate from the holoendemic Lake Victoria area and travel back and forth intermittently to their home areas; however, this travel pattern has been occurring since the road was surfaced in the 1950s and has not changed recently. This study was conducted under a protocol approved by the Kenyan National Ethical Review Committee (SSC 484) and the U.S. Army Office of the Surgeon General (WRAIR 682).

## Meteorologic Data

Two meteorologic datasets were compiled. Point locality measurements of mean monthly temperature (°C) and monthly total rainfall (mm) were obtained from the Tea Research Foundation meteorologic station on the Kericho tea estates for the 1966–1995 period. Climate data were also obtained from a global 0.5 x 0.5° (approximately 55 x 55 km [3,025 km^2^] at the equator) gridded dataset of monthly terrestrial surface climate for the 1966–1995 period (33,34) (available from: URL: http://www.cru.uea.ac.uk/link). The dataset was used to ensure that results from the single meteorologic station were in agreement with data from a wider geographic area; this procedure also allowed a wider range of climate variables, including temperature extremes, to be tested. Primary variables of precipitation (mm), mean temperature (°C), and diurnal temperature range (°C) were available and interpolated from extensive meteorologic station data by using angular distance-weighted averaging of anomaly fields. The secondary variable of vapor pressure was also provided, interpolated where available, and calculated from primary variables, when the coverage of meteorologic stations was insufficient. Minimum and maximum monthly temperature estimates were created by subtracting or adding, respectively, half the diurnal temperature range from mean monthly temperature. Time series were derived by using an extraction routine developed in ENVI (Research Systems Inc., Boulder, CO) with georeferencing information for Kericho (0.33°S, 35.37°E), obtained from Encarta (Microsoft, Seattle, WA).

To investigate whether a combination of meteorologic conditions was changing and thus facilitating the resurgence of malaria, we also categorized months as suitable for *Plasmodium falciparum* transmission if they had a mean monthly temperature exceeding 15°C (since temperatures experienced by the indoor resting *Anopheles gambiae* vectors are likely to be 3°C–5°C higher) and monthly rainfall totals exceeding 152 mm (1,4) by using the gridded climatology data. The numbers of suitable months for transmission were summed, totaled for each year, and tested for the 1966–1995 period.

### Statistical Analyses

To test for trends in the climate and malaria suitability time series, we estimated the following regression equation:Δy_t_ = α + β t + γ y_t-1_ + 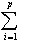
δ_i_ Δy_t-1_ + 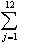
μ_j_ d_j_ + ε_t_
(1)where y is the variable of interest; α, β, γ, and μ_j_ are regression parameters; ε_t_ is a normally distributed error term with mean zero; and t is a deterministic time trend. The centered dummy variables d_j_ model the monthly seasonal variations in climate. The coefficients μ_j_ sum to zero. Δ is the first difference operator. The lagged values of the dependent variable model the serial correlation in the dependent variable. We chose the number of lags, p, using the adjusted R-square statistic. The maximal number of lags p considered was 24.

If the time series y can be characterized as the sum of a stationary stochastic process and a linear time trend, then the appropriate test for the trend is a t test on β in (1). If the series is a random walk, however, or a more complex stochastically trending process, the critical levels for the distribution of the t score in this regression are much greater than usual (35), and alternative tests should be employed. Since many climate time series contain a stochastically trending component (36), the nature of the series must be explored before testing for climate change. This methodologic issue complicates the evaluation of the significance of trends established with standard regression procedures often used in such studies.

If γ =0 (a unit root in the autoregressive process) and β =0, then y is a random walk. The random walk may also have a deterministic drift term (α≠0). In either case, however, the series is nonstationary, and classical regression inference does not apply. The nonstandard distributions of α, β, and γ have been tabulated by Dickey and Fuller (37,38). We first tested for the presence of a unit root by evaluating the t statistic for γ against its nonstandard distribution. The critical value for this so-called Augmented Dickey-Fuller at the 5% level is -3.45. Values of the t statistic for γ more negative than this critical value indicate that the series is not a random walk and vice versa. If the null hypothesis is rejected, then the t statistics associated with α and β are normally distributed. If the unit root hypothesis is accepted, then these statistics also have nonstandard distributions. The correct test for a trend is then the t test on α in (1) with the omission of the linear trend. The test’s critical value at the 5% significance level is 2.54. The results of these tests are presented in the Table.

**Table:** Trend of malaria, climate, and malaria suitability variables, Kericho tea estates, 1966–1995^a,b^

Variable	p	ADF^c^	β	t	p value^c^	τα	Q	Sig. Q
Malaria incidence	5	**-4.00**	0.0238	**2.49**	0.0133	0.1801	58.7394	0.0097
Total admissions	6	-2.76	-0.0069	-0.28	0.7820	-0.4151	30.9302	0.7083
Tmean met. stat. (^o^C)	8	-3.41	0.0004	1.76	0.0799	-0.0211	40.8630	0.2653
Rain met. stat. (mm)	1	**-11.91**	-0.0202	-0.52	0.6066	-0.0074	43.3753	0.1858
Tmean clim. (^o^C)	1	**-7.51**	0.0035	1.60	0.1103	-0.0980	46.6888	0.1094
Tmax clim. (^o^C)	24	**-4.66**	0.0070	1.68	0.0935	0.0592	22.6634	0.9592
Tmin clim. (^o^C)	1	**-8.36**	0.0038	1.55	0.1233	-0.1944	45.1424	0.1412
Precipitation clim. (mm)	1	**-11.70**	-0.0098	-0.36	0.7205	-0.0745	34.2984	0.5497
Vapor pressure clim. (hPa)	1	**-8.37**	0.0038	1.66	0.0974	-0.1829	45.5674	0.1318
Garnham suitability (mo)^d^	4	**-4.21**	-0.0380	-**0.89**	0.3850	-0.4488	5.6658	0.7729

^a^Tmean, the mean monthly temperature; Tmax, the mean of maximum monthly temperatures; Tmin, the mean of minimum monthly temperatures; met. stat., meteorologic station data from the Kericho tea estate; clim., data derived from the global gridded climatology dataset (33,34). ^b^Figures in bold denote significance at the 5% level. p is the number of lagged differenced dependent variables selected. ^c^ADF, the Augmented Dicke-Fuller t-test for γ=0. The 5% critical value is -3.45. Exact p values are not available for ADF and τα statistics. The distribution of the t statistic for the slope parameter β has the standard t distribution under the assumption that γ<0. τα is the t statistic for the intercept term in the autoregression without a linear time trend. This test is the appropriate one for a trend if γ=0. Its 5% critical value is 2.54. The Q statistic is a portmanteau test for general serial correlation and is distributed as chi square (39). ^d^Garnham suitability (1,4) refers to the number of months with a mean monthly temperature exceeding 15°C and monthly rainfall totals exceeding 152 mm (when the gridded climatology data are used). These data are therefore annual data, whereas all other time-series are monthly observations.

We also regressed temperature and rainfall data from the meteorologic station at Kericho on the same variables from the interpolated climatology (33,34) by using a variety of formulations including levels, logarithms, and a regression adjusted for heteroscedasticity. We then tested whether the slope coefficients were significantly different from unity, which should not be the case if the gridded dataset is a good proxy for the climate at Kericho.

## Results

**Figure 1:**
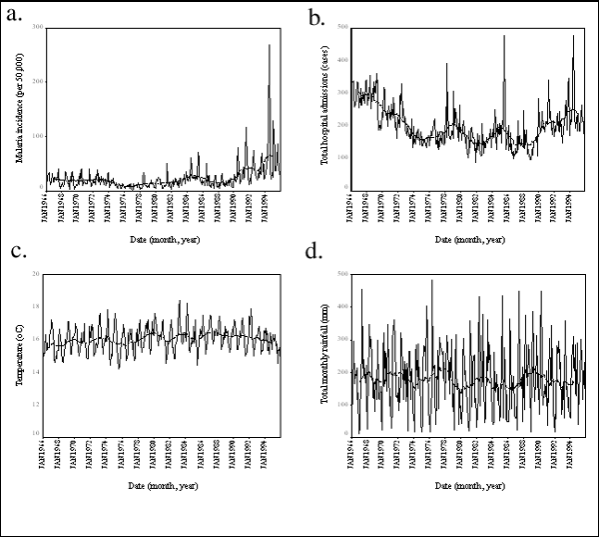
Malaria, hospital admissions, and meteorologic station data, Kericho tea estate, 1966–1995. Malaria incidence (a) total hospital admissions (b) mean monthly temperature (c) and total monthly rainfall (d) are all plotted with a 25-point (month) moving average (bold) to show the overall movement in the data. The significance of these movements is presented in Table.

**Figure 2:**
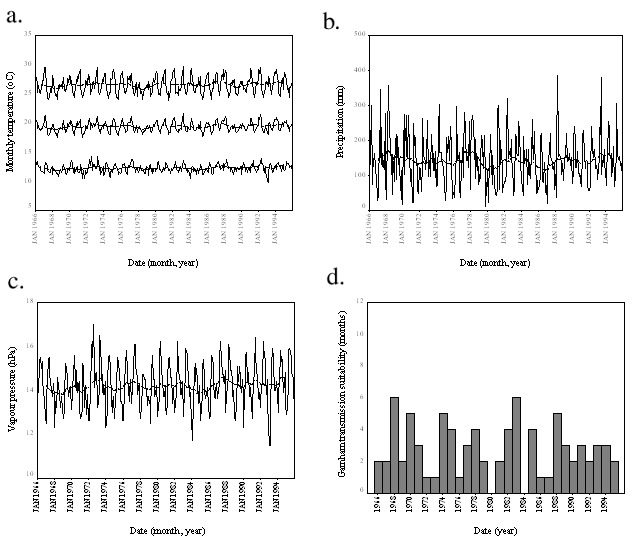
Climate and malaria suitability data for the Kericho area from the global gridded climatology data, including meteorologic and malaria suitability time series. Minimum (bottom), mean (middle) and maximum (top) monthly temperature (a) total monthly precipitation (b) and mean vapor pressure (c) are all plotted with a 25-point (month) moving average (bold) to show the overall movement in the data. The number of months per year suitable for malaria transmission (d) are also plotted. Suitability was determined if rainfall exceeded 152 mm and temperature exceeded 15°C in any month (1,4). The significance of these movements is presented in Table.

During the period 1966–1995, malaria incidence increased significantly (p=0.0133) while total (i.e., malarial and other) admissions to the tea estate hospital showed no significant change (Table and Figure 1a,b). Measurements of mean monthly temperature and total monthly rainfall also showed no significant changes (Table and Figure 1c,d). Similar results were shown by the climatology data interpolated from a wider area. Mean, maximum, and minimum monthly temperatures; precipitation; and vapor pressure all demonstrated no significant trends (Table; Figure 2a,b,c). Moreover, the interpolated climatology data, when transformed into month of malaria transmission suitability (1,4), again showed no significant changes (Table; and Figure 2d).

Results were very similar, though significance levels varied, between the three formulations of the regression model that compared the local meteorologic station data and those from the interpolated climatology data (33,34). The coefficient for the regression of the meteorologic station rainfall data on the interpolated climatology precipitation data is in every case not significantly different from unity. Significance levels are 10% for the model in levels, 18% for the heteroscedasticity-adjusted model, and 96% for the logarithmic model. In the regression of the two temperature series, however, the coefficient is significantly different from unity in every case, as is a joint test statistic for the two slope coefficients.

## Discussion

The resurgence of *P. falciparum* malaria in the East African highlands (3,8,18,26, 40–44) has led several researchers to speculate that climate change is a predominant cause (23,45–50). On the basis of these studies, which have been disputed by experts in vector-borne disease biology (10,27–29, 51,52), and some biological modeling, which has been robustly criticized (53), the International Panel on Climate Change has recently concluded with “medium-to-high confidence” that there will be a net increase in the range and incidence of malaria (49)**;** the results of our work do not support these conclusions.

Malaria incidence increased significantly (p=0.0133) during the 1966–1995 period, while total admissions remained unchanged. Besides an increase in local malaria transmission, two other factors may have influenced the increase in malaria hospitalizations. An increase in malaria severity indicated by an increased case-fatality rate (from 1.3% in the 1960s to 6% in the 1990s) is most likely linked to chloroquine resistance, which we believe to be the probable cause of much of the overall increase in malaria transmission (32). Travel to and from the Lake Victoria region by a minority of the tea estate workers also exerts an upward influence on malaria transmission in Kericho since such travel increases the numbers of workers asymptomatically carrying gametocytes, which infect mosquitoes for further human infection. This complex topic is the subject of a future publication.

All climate variables, whether from the Kericho tea estate meteorologic station or the pixel covering Kericho in the global climatology dataset showed no significant trends, despite the fact that equivalence tests showed some significant differences between the temperature time series—findings that are in agreement with a broader geographic analysis of East African data from 1911 to 1995 (26) and lend support to the appropriateness of interpolated climate data for use in these investigations. We also think that, when examining trends in meteorologic phenomena, epidemiologists should use more robust statistical techniques for the reasons outlined in the methods. The results of this detailed examination of coincident empirical data do not support the widespread, recent speculation regarding malaria resurgences in response to climate change. No aspect of climate has changed significantly—neither the temperature extremes (maximum and minimum) nor the periods when meteorologic data were transformed into months when malaria transmission is possible. Further study has also shown that variability in these meteorologic variables, independent of any longer term trends, has decreased (54). We must therefore look elsewhere for the causes of these resurgences (27,28,32). These factors are likely to vary. In Kericho, however, increased chloroquine resistance has been strongly argued to be the cause, since all other relevant environmental and sociologic factors are unchanged (32).

The attraction of the global warming hypothesis as an explanation of highland malaria is the existence of a continental trend toward global warming coincident with a trend toward increasing malaria incidence in several parts of Africa, ranging from Senegal (13,14) to Madagascar (10). Where such malaria increases have been examined in detail, however, alternative explanations such as discontinuation of anti-vector measures in Madagascar (10) or chloroquine resistance in Senegal appear to be more likely causes (13,14). Malaria epidemiology is greatly influenced by a range of local factors, making a consistent continent-wide explanation seem unlikely (28,52).

We do not argue that meteorologic conditions have no immediate impact on the seasonal dynamics and incidence of malaria or that climate change is probably not an important future concern in public health. Rather we urge some caution in the interpretation of synonymous changes in climate over wider areas and local changes in malaria incidence.
